# Otology

**Published:** 1895-12

**Authors:** W. H. Harsant


### OTOLOGY.

Professor Urbantschitsch, of Vienna, has lately drawn attention [2] to a new method of developing the hearing power of deaf-mute children by a systematic course of acoustic gymnastics. He has now given this method a thorough trial, and has been much surprised and gratified by the results obtained. He begins by practising the simplest voice sounds in one ear of the child for five or ten minutes every day. He next practises on the other ear, proceeding gradually to simple words and then to sentences. After a little the patient is exercised in other voices, because he found that deaf-mutes could only at first understand

[2] *Wien. klin. Wchnschr.*, 1893, vi. 525, and 1894, vii. 343, 368.

one person. This is in accordance with the point raised by Bezold that some deaf-mutes are without hearing for certain groups of sound, of greater or less extent. When patients hear a certain group of sounds he gradually extends this in both directions. He then practises with tuning-forks, and especially with a small instrument of five and a half octaves, which is a kind of harmonium. This is valuable because the sound lasts longer than that of a tuning-fork. He begins with one note of the harmonium, and then presses down two notes together, in order that the patient may learn to differentiate tones. These practices are to be continued until the sense of hearing is so developed that speech is understood. If the exercises be interrupted the patient sinks back and relapses, but he can practise by himself with the harmonium. Urbantschitsch has found a total word deafness to be rare in deaf-mutes. Even in those patients who appear so at first, a certain hearing of one tone may be found with patient care, and from this he may be led on first to a vowel sound and then to words. This may take place more quickly in one ear than in the other. Some patients cannot be got beyond the first stage, but others may be led on further. The mental condition of the patient has great influence. If this is very bad we must first raise the mental level by some other means of education. When the patient has got to words it is important to practise with those words to which he attaches a definite meaning, such as those things which he is habitually handling, as fork, spoon, &c. Professor Urbantschitsch records some cases illustrating the results of his method, such as a girl, nine years old, who was totally deaf from birth, but after she had practised this method four times a week for ten months could hear words and put together easy sentences; also a boy, aged fifteen, who became totally deaf in his fifth year from meningitis; he practised these exercises daily for ten minutes, and in six months he could understand all sounds and could hear words and sentences and his own voice.

Dr. J. Steele Barnes, of Milwaukee, publishes an interesting article on the same subject,[1] in which he points out that the idea of aural gymnastics in the treatment of deaf-mutism did not originate with Professor Urbantschitsch, but, so far as we know, with Bewus[2] over a century and a half ago. Later, Itard[3] in 1821, and Toynbee[4] in 1860, reported success with cases treated by similar methods; but to Urbantschitsch we are indebted for developing the idea and proving its great value. His attention was called to what was possible of accomplishment with the method by his remarkable success in the case of a deaf-mute boy. He says:[5] "Several years ago I obtained a surprising improvement of hearing by systematic acoustic

[1] *Ann. Ophth. & Otol.*, 1895, iv. 390.
[2] Beck. *Ohrenheilkunde*, 1827, p. 73.
[3] *Traité des Maladies de l'Oreille*, ii. 476. [4] *Diseases of the Ear.*
[5] *Wien. klin. Wchnschr.*, 1893, vi. 525.

gymnastics in the case of a deaf-mute boy referred to me for treatment. At first he was able to hear only vowels and single syllables spoken loudly in his ear; later, after a year, he could understand complete sentences spoken in a moderately loud voice and finally was able to receive ordinary, not deaf-mute instruction." There is nothing extraordinary or intricate in the method. It is virtually a vocal training of the auditory nerve, whereby a gradual irritability of the nerve for sound waves is set up. It is, as it were, a bringing into life and activity a hitherto lifeless nerve. At the Vienna Policlinic, Professor Urbantschitsch devotes an hour daily to the training of deaf-mutes by acoustic gymnastics, and it was there Dr. Barnes's good fortune to receive personal instruction and witness the surprising results obtained. It was indeed interesting to note the gradually increasing perception of sound in ears that were at first apparently totally deaf. Instruction begins with the vowels. Two are selected, usually A and O, as they are the most readily understood. The one to be used is first indicated to the patient by the lips, and then in a loud and prolonged voice is spoken directly into the ear. The patient in the beginning may not hear a sound, but persistent and continued effort will, even sometimes at the first sitting, enable him to arrive at a differentiation of two sounds. While it may not be a complete differentiation, the sound of A conveys a different impression to the mind than the sound of E as shown by the effort of the patient to repeat them. Again, this result may not be obtained until after several sittings. As soon as the differentiation of the vowels is mastered consonants are added to them, gradually increasing to long syllables, then to words, and finally complete sentences. As progress is made the distance from the ear at which the exercise is conducted may also be gradually increased, and after a few sittings it is best to dispense with the lip method, making the exercise purely acoustic.

While perception of sound is essentially necessary to gaining any progress in a case, conception of sound makes the perception easier, exerting a marked influence upon the rapidity of improvement. One may have perception long before he has any conception of a sound, but the sooner a sound conveys intelligence to the brain the quicker and more readily is that sound always distinguished. Therefore, from the very beginning the attempt should be made to convey to the patient a meaning with every sound, to have him realise that every sound has an intelligent expression for the mind. Regarding the duration and frequency of the sittings, Urbantschitsch has found that short, continued practising of from five to ten minutes gives the best results. Practice should be daily at first, gradually increasing in frequency rather than lengthening the duration.

Dr. M. A. Goldstein, of Saint Louis, has an article[1] very

[1] *Arch. Otol.*, 1895, xxiv. 50.

warmly praising the method, and describing what he has personally witnessed of the marked improvement taking place from month to month. He has found that beneficial results from the use of this system are equally distributed in cases of *congenital* or *acquired* deafness. *Age* also seems to offer no obstacle to success. In the deaf-mute asylums of Austria practical tests have yielded most gratifying results. For nearly one year these exercises have been combined with the regular system of instruction, and the progress of the pupils, as indicated by recent tabulated reports, has been remarkable. A number of these pupils, to whom the special instruction had been given, were presented before the Society of Physicians, in Vienna, April 27th last, and the fact that these congenitally deaf pupils could hear the speaker at distances varying from several inches to ten feet, without the aid of lip-reading or signs, was in itself the most brilliant testimony of success.

W. H. Harsant.

